# Real-world intravascular ultrasound (IVUS) use in percutaneous intervention-naïve patients is determined predominantly by operator, patient, and lesion characteristics

**DOI:** 10.3389/fcvm.2022.974161

**Published:** 2022-11-08

**Authors:** Alexander Tindale, Vasileios Panoulas

**Affiliations:** ^1^Royal Brompton and Harefield Hospitals, Guy’s and St Thomas’ NHS Foundation Trust, London, United Kingdom; ^2^National Heart and Lung Institute, Imperial College London, London, United Kingdom

**Keywords:** IVUS (intravascular ultrasound), PCI—percutaneous coronary intervention, real-world, regression analysis, propensity matching

## Abstract

**Background:**

Intravascular Ultrasound (IVUS) has been shown to improve clinical outcomes in patients undergoing percutaneous intervention (PCI) in numerous trials. However, it is still underutilized outside of trial settings, and most trials include a significant proportion of patients with prior PCI. The aim of this study is to look at real-world use and outcomes in PCI-naïve patients who undergo IVUS-guided intervention.

**Methods and results:**

Prospectively collected data from 10,574 consecutive patients undergoing their index PCI was retrospectively analyzed. 455 (4.3%) patients underwent IVUS, with a median follow-up of 4.6 years. Patients undergoing IVUS had higher levels of comorbidities including diabetes (27.5% vs. 19.7%, *p* < 0.001), hypertension (58.0% vs. 47.9%, *p* < 0.001), hypercholesterolemia (51.6% vs. 39.2%, *p* < 0.001) and were generally older (65.9 ± 14.5 vs. 64.5 ± 13.4 years, *p* = 0.031) with higher mean baseline creatinine levels (95.4 ± 63.3 vs. 87.8 ± 46.1 μmol/L). The strongest predictor of IVUS use was the operating consultant graduating from medical school after the year 2000 [OR 14.5 (3.5–59.8), *p* < 0.001] and the presence of calcific lesions [OR 5.2 (3.4–8.0) *p* < 0.001]. There was no significant difference in MACE nor 1-year mortality between patients undergoing IVUS-guided or angiography-only PCI on unadjusted analysis [OR 1.04 (0.73—1.5), *p* = 0.81, OR 1.055 (0.65–1.71) *p* = 0.828] nor mortality throughout the study period (HR 0.93 (0.69—1.26), *p* = 0.638). This held true for stents longer than 28 mm. Propensity matched analysis of patients similarly showed no mortality difference between arms for all patients and those with longer stents (*p* = 0.564 and *p* = 0.919).

**Conclusion:**

The strongest predictors of IVUS use in PCI-naïve patients are the operator’s year of graduation from medical school and proxy measures of calcific lesions. On both matched and adjusted analysis there was no evidence of improved mortality nor reduced MACE in this specific retrospective cohort, although this may well be explained by significant selection bias.

## Introduction

The use of intravascular ultrasound (IVUS) has steadily increased since its development in the late 1980s (1), and this use was accelerated by the evolution of effective drug-eluting stents (DES) a decade later (2). Subsequently, there have been numerous large-scale randomized trials assessing the effectiveness of IVUS in the drug-eluting stent era.

In general, these show that IVUS-guided PCI reduces major adverse cardiac events (MACE) in a variety of different settings. These include complex coronary artery disease (3), all-comers with follow-up periods of up to 3 years (4) and patients requiring stents longer than 28 mm (5, 6). The largest synthesis looked at 31 studies, including both randomized and observational trials, and demonstrated that MACE was lower with IVUS use with the suggestion of mortality benefit when including observational data (7).

As a result of this evidence, IVUS has grown in popularity and is now a class IIa recommendation in the latest ESC guidelines (8). However, outside of trials, IVUS use remains heterogenous and often low. For example, IVUS is used in 12% of PCI cases in the UK (9), but 80% of such cases in Japan (10).

Furthermore, a significant proportion of patients in these trials have undergone previous revascularization, ranging from 11 to 48% (11). There is limited literature on the effect of IVUS in PCI-naïve patients.

Therefore, the aims of this study are threefold. Firstly, we begin with a descriptive analysis to examine which patient, procedural and operator characteristics are associated with IVUS use in PCI-naïve patients, to shed light on the relative underuse of IVUS.

Secondly, we analyze the clinical outcomes of all PCI-naïve patients who received second generation drug-eluting stents of any length with and without IVUS optimization.

Thirdly, in light of the evidence from a recent analysis concerning IVUS effectiveness in long stents, we perform subgroup analysis for patients with implanted stents of longer than 28 mm.

## Materials and methods

### Study population and design

**FIGURE 1:**
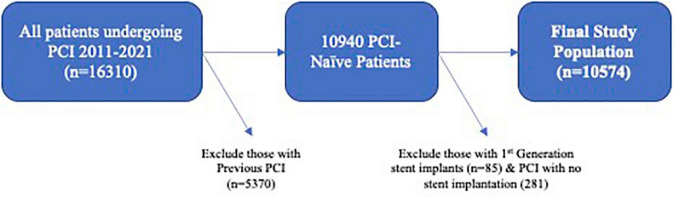
Study flow-chart.

This was an observational study to determine associations between patient and operator characteristics and IVUS use, in addition to associations between IVUS use and favorable clinical outcomes. The study population was 10,574 consecutive patients undergoing their first PCI with second-generation stent implantation at Harefield Hospital between January 1st 2011 and January 1st 2021. The study flow-chart is shown in Figure 1. Patients who underwent optical coherence tomography (OCT) were excluded.

### Clinical and outcome data

The majority of clinical data, including patient characteristics and comorbidities, were taken from the audit data collected for every PCI patient at our institution. Blood gas analysis, hematology tests and laboratory biochemistry tests were taken from our own hospital’s database. Health outcomes data including survival was obtained by linking patients’ NHS numbers to the NHS spine, in collaboration with the Office for National Statistics (ONS).

The primary outcome measures were 1-year mortality and 1-year MACE, which is a patient-oriented composite endpoint encompassing all-cause mortality, unplanned revascularization, stroke and myocardial infarction (MI). Secondary outcome measures were a composite of device endpoints that includes cardiovascular (CV) death, MI and target-lesion revascularization (TLR). These were chosen in line with the Academic Research Consortium’s (ARC) guidelines for outcome reporting in cardiovascular trials (12). When the cause of death was unknown, it was listed as a cardiovascular death for the purposes of analysis in line with the ARC guidance.

### Ethics

All patient-identifiable information was removed before analysis. Our local audit office assigned institutional support for this project. As this was analysis of anonymized information taken from required audit data we were advised that no further ethical approval was required.

### Statistical analysis

Univariate analysis was performed using Student’s *t*-test for comparing the means of normally distributed data and Mann-Whitney U if not normally distributed. Chi-squared and Fisher’s tests were used for categorical data. Fisher’s exact test was used if the expected value in any group was less than five. Regression analysis was performed using binary logistic regression for dichotomous outcome variables and cox proportional hazards and Kaplan-Meier curves for survival data, as appropriate. These tests were performed in *SPSS* (IBM SPSS Statistics, version 28 (IBM Corp., Armonk, N.Y., USA) and *R* (R Foundation for Statistical Computing, Vienna, Austria).

Propensity matching was performed using *R*. The method used was 1:1 nearest neighbor matching without replacement, where distance was defined by using a propensity score estimated by logistic regression. The covariates used for propensity matching were age, ACS, previous ACS, previous CABG, hypercholesterolemia, smoking status, diabetes, hypertension, cardiac arrest, out of hospital cardiac arrest (OOHCA), maximum stent length per vessel, devices used for calcific lesions, devices used for calcium modification, number of stents used, year of procedure, hemoglobin, white blood cell concentration, sodium, potassium, urea, creatinine, consultant age band and consultant operator.

Statistical significance was established at *p* < 0.05 (2-tailed) for all tests. All data is reported according to the STrengthening the Reporting of OBservational studies in Epidemiology (STROBE) guidelines (13).

## Results

### The general landscape of intravascular ultrasound use

In total, 10,574 PCI-naïve patients underwent an initial percutaneous intervention at Harefield Hospital with second generation DES implantation. The full baseline characteristics can be seen in Table 1. Notably 75.3% of procedures were for acute coronary syndromes (ACS), and of these, 77% were treated for ST elevation myocardial infarction (STEMI).

**TABLE 1:** Baseline characteristics and univariate analysis of patients undergoing index PCI 2011–202.

	All patients (*n* = 10,574)	IVUS not used (*n* = 10,119)	IVUS used (*n* = 455)	*P*
**Patient characteristics**				
Male sex	7,825 (74.0)	7,746 (74.0)	351 (74.7)	0.544
Age (years)	64.5 (13.4)	64.5 (13.4)	65.9 (14.5)	0.031
Weight (Kg)	83.2 (127.3)	84.0 (146.6)	80.7 (18.0)	0.67
Systolic BP	133.2 (188)	130.9 (155.9)	129.2 (28.5)	0.884
Previous ACS	1,105 (10.5)	1,008 (10.0)	97 (21.3)	< 0.001
Previous CABG	630 (6.0)	587 (5.8)	43 (9.5)	0.001
Hypercholesterolemia	4,150 (39.2)	3,915 (38.7)	235 (51.6)	< 0.001
Current smoker	2,491 (23.6)	2,431 (24.0)	60 (13.2)	< 0.001
Ex-Smoker	2,696 (25.5)	2,536 (25.1)	160 (35.2)	< 0.001
Diabetes	2,121 (20.1)	1,996 (19.7)	125 (27.5)	< 0.001
HTN	5,115 (48.4)	4,851 (47.9)	264 (58.0)	< 0.001
**Procedure details**				
ACS	7,961 (75.3)	7,733 (76.4)	228 (50.1)	< 0.001
STEMI	6,132 (58.0)	6,007 (59.4)	125 (27.5)	< 0.001
NSTEMI	1,829 (17.3)	1,726 (17.0)	103 (22.6)	< 0.001
Cardiac arrest	808 (7.6)	786 (7.8)	22 (4.8)	0.021
OOHCA	483 (4.6)	472 (4.7)	11 (2.4)	0.025
Longest stented/treated segment	24.8 (10.8)	23.4 (27.5)	27.5 (14.8)	< 0.001
Max stent length per vessel	25.8 (16.2)	24.6 (14.9)	30.0 (19.8)	< 0.001
Max balloon diameter	4.7 (23.5)			0.501
Number stents used	1.5 (0.9)	1.4 (0.9)	2.1 (1.4)	< 0.001
Devices for calcium	467 (4.4)	357 (3.5)	110 (24.2)	< 0.001
Calcium modification only	360 (3.4)	269 (2.7)	91 (20)	< 0.001
**Vessels treated**				
PCI LMS	393 (3.7)	374 (3.7)	19 (4.2)	0.597
PCI LAD	4,656 (44.0)	4,463 (44.1)	193 (42.4)	0.478
PCI LCx	2,207 (20.9)	2,102 (20.8)	105 (23.1)	0.237
PCI RCA	3,277 (31.0)	2,135 (31.0)	142 (31.2)	0.918
PCI grafts	158 (1.5)	153 (1.5)	5 (1.1)	0.690
**Blood gas at time of procedure**				
pH	7.4 (0.1)	7.40 (0.08)	7.41 (0.07)	0.071
BE	-1.2 (4.2)	-1.7 (4.4)	-0.3 (4.6)	< 0.001
HCO3	23.6 (3.1)	23.1 (3.4)	24.1 (3.4)	< 0.001
Lactate	2.1 (1.9)	2.3 (2.4)	1.8 (1.7)	< 0.001
Glucose BG	8.6 (3.5)	8.7 (3.7)	8.6 (3.5)	0.549
**Laboratory blood values**				
Hb	120.0 (43.6)	113 (49.6)	119.7 (38.8)	0.004
WCC	11.0 (5.0)	11.1 (4.7)	10.0 (6.9)	< 0.001
Sodium	136.0 (3.6)	136.0 (3.5)	136.1 (3.9)	0.531
Potassium	4.1 (0.5)	4.1 (0.5)	4.2 (0.4)	0.002
Urea	6.6 (3.4)	6.6 (3.2)	7.3 (4.1)	< 0.001
Creatinine	86.5 (44.0)	87.8 (46.1)	95.4 (63.3)	< 0.001
Bilirubin	12.0 (6.9)	12.0 (6.6)	12.8 (8.4)	0.025
ALT	51.0 (134.6)	49.3 (128.7)	54.9 (149.5)	0.416
Albumin	38.6 (5.0)	38.6 (5.1)	38.5 (5.3)	0.775
ALP	79.6 (37.3)	78.4 (35.0)	78.6 (5.3)	0.048
CRP	19.6 (41.8)	18.6 (40.8)	26.1 (50.6)	0.004
Trop I ng/L	14463.6 (27833.9)	14233.8 (24746)	15112.3 (35030.8)	0.652
Mg	0.8 (0.1)	0.8 (0.1)	0.8 (0.1)	0.316
Cholesterol	4.9 (1.3)	4.9 (1.3)	4.6 (1.3)	0.002
**Operator characteristics**				
Consultant 1st operator	6,985 (66.1)	6,636 (65.6)	349 (76.7)	< 0.001
Operator’s year of qualification				< 0.001
Before 1990	2,502 (23.7)	2,473 (24.4)	29 (6.4)	
1990–1999	5,838 (55.2)	5,588 (55.2)	250 (54.9)	
Post 2000	2,234 (21.1)	2,058 (20.3)	176 (38.7)	
Operator’s case numbers	1510.7 (1004.2)	1492.0 (998.1)	1928.9 (1047)	< 0.001

**FIGURE 2:**
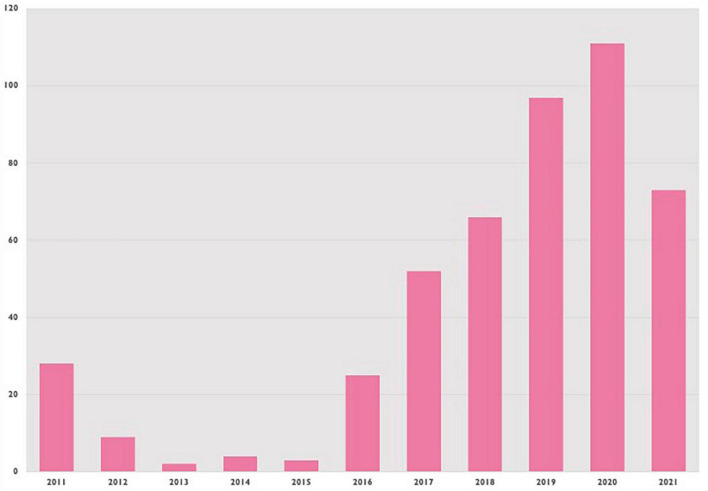
Absolute number of IVUS procedures performed in PCI-naive patients by year.

IVUS was used in 455 patients, or 4.3% of cases. Figure 2 shows IVUS procedures (for all patients) over time, showing a general trend upwards since 2011. Median follow-up for all included patients was 1,691 days (IQR 753–2796).

### Univariate analysis showing associations with intravascular ultrasound use

Univariate analysis of different patient and procedural characteristics are show in Table 1. IVUS use was more prevalent in chronic rather than ACS: 50.1% of patients in the IVUS group were treated for ACS compared to 76.4% in the non-IVUS group (*p* < 0.001). However, the IVUS group had significantly higher rates of diabetes (27.5% vs. 19.7%, *p* < 0.001), hypertension (58.0% vs. 47.9%), hypercholesterolemia (51.6% vs. 38.7%, *p* < 0.001) and were generally older (65.9 ± 14.5 vs. 64.5 ± 13.4 years, *p* = 0.031) with higher mean baseline creatinine levels (95.4 ± 63.3 vs. 87.8 ± 46.1 μmol/L).

In contrast, IVUS use was less prevalent in patients with markers of acute illness, with lower prevalence in patients with STEMIs and cardiac arrests. Related to this, patients undergoing IVUS-guided PCI had significantly lower mean lactate levels than those without IVUS guidance (1.8 ± 1.7 vs. 2.3 ± 2.4 mmols/L, *p* < 0.001).

Moreover, both longer length and higher complexity of coronary lesions were associated with choice of IVUS use, but there was no significant difference in the which vessel was treated nor vessel diameter between groups. The mean length of implanted stent per vessel was 30.0 ± 19.8 mm in the IVUS group vs. 24.6 (14.6) mm in the non-IVUS group (*p* < 0.001). Furthermore, patients undergoing IVUS had a higher mean numbers of stents (2.1 ± 1.4 vs. 1.4 ± 0.9, *p* < 0.001) and significantly higher rates of concomitant calcium modification device use. These include intracoronary laser, rotational atherectomy and shockwave lithotripsy: 19.8% of patients in the IVUS group had concomitant calcium modification vs. 2.6% in the non-IVUS group (*p* < 0.001). The variable “devices for calcium” was deemed positive if any calcium modification device or other devices that may be used in calcific lesions such as microcatheters.

Finally, there were large differences in the operator characteristics, with IVUS being favored heavily by more recently qualified consultants, discussed in the multivariate analysis section below.

### Multivariate analysis of factors associated with intravascular ultrasound use

The largest single predictor of IVUS use was the year that the operating consultant graduated from medical school (Table 2). Consultants graduating after 2000 were almost 15 times more likely to use IVUS than those graduating before 1990, even adjusting for the year that the procedure was performed (OR 14.5 (3.5–59.8), *p* < 0.001). The next most powerful predictor was the presence of calcific lesions, which led to a five-fold increase in the odds of IVUS use [OR 5.2 (3.4–8.0) *p* < 0.001]. The presence of STEMI made IVUS use around a third as likely [OR 0.34 (0.23–0.49), *p* < 0.001].

**TABLE 2:** Multivariate analysis of IVUS use.

Variable	*P*	*OR*	Lower 95% CI	Upper 95% CI
Age (years)	0.046	0.987	0.974	1
Prev CABG	0.036	0.573	0.341	0.963
Calcific Lesions	< 0.001	5.223	3.383	8.064
STEMI	< 0.001	0.337	0.234	0.485
Qualified before 1990	< 0.001			
Qualified 1990–1999	0.026	5.397	1.227	23.735
Qualified 2000-	< 0.001	14.493	3.509	59.865
Operator case number	< 0.001	1.001	1	1.001
Year	0.005	1.101	1.03	1.178
Max stent length per vessel (mm)	0.012	0.987	0.977	0.997
Number stents used	< 0.001	1.593	1.374	1.847
Constant	0.004	0		

### Unadjusted and adjusted outcomes of intravascular ultrasound use

**FIGURE 3:**
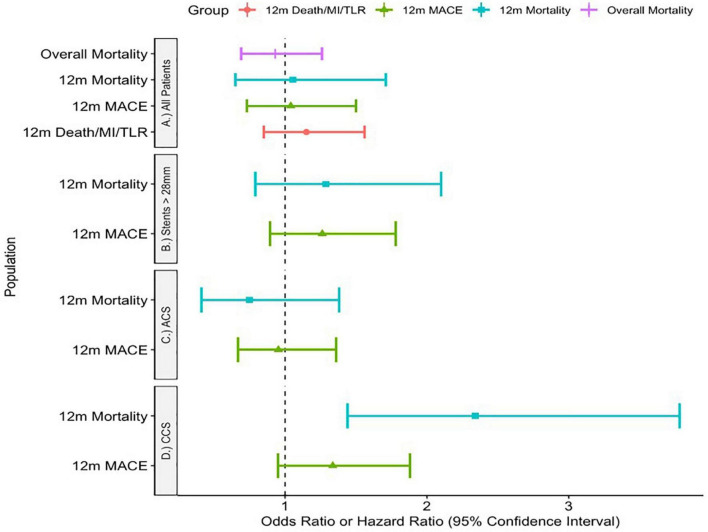
Adjusted outcome measures.

Unadjusted outcomes are listed in Table 3. Adjusted outcomes are shown visually in Figure 3.

**TABLE 3:** Unadjusted outcomes.

Outcome				

All patients	All patients (*n* = 10,574)	IVUS not used (*n* = 10,119)	IVUS used (*n* = 455)	*P*
Composite death/Stroke/MI/Unplanned revasc 1 year	2,058 (19.5)	1,963 (19.4)	95 (20.9)	0.436
Death 1 year	903 (8.5)	855 (8.4)	48 (10.5)	0.117
Stroke 1 year	8 (0.1)	8 (0.1)	0 (0)	0.549
MI 1 year	300 (2.8)	286 (2.8)	15 (3.3)	0.546
Unplanned revasc 1 year	1,000 (9.5)	961 (9.5)	39 (8.6)	0.509
Composite CV death/MI/TLR 1 year	1,241 (11.7)	1,181 (11.7)	60 (13.2)	0.326
TLR	262 (3.4)	352 (3.5)	10 (2.2)	0.142
Stent thrombosis	84 (0.8)	78 (0.8)	6 (1.3)	0.198

**Stents longer than 28 mm**	**All patients (*n* = 3,592)**	**IVUS not used (*n* = 3,370)**	**IVUS Used (*n* = 222)**	***P***

Composite death/Stroke/MI/Unplanned revasc 1 year	704 (19.6)	650 (19.3)	54 (24.3)	0.067
Death 1 year	304 (8.5)	279 (8.3)	25 (11.3)	0.122
Stroke 1 year	0 (0)	0 (0)	0 (0)	1
MI 1 year	101 (2.8)	94 (2.8)	7 (3.2)	0.751
Unplanned revasc 1 year	348 (9.7)	321 (9.5)	27 (12.2)	0.198
Composite CV death/MI/TLR	419 (11.7)	390 (11.6)	29 (13.1)	0.503
TLR	109 (3)	104 (3.1)	5 (2.3)	0.684
Stent thrombosis	40 (1.1)	38 (1.1)	2 (0.9)	1

Adjusting for significant comorbidities, there was no significant difference in MACE at 1 year using IVUS [OR 1.04 (0.73—1.5), *p* = 0.81], 1-year mortality [OR 1.055 (0.65–1.71) *p* = 0.828] nor mortality throughout the study period [HR 0.93 (0.69—1.26), *p* = 0.638]. Furthermore, there was no difference in the device endpoint of MI/Death/TLR at 1 year [OR 1.15 (0.85–1.56) *p* = 0.361].

In chronic coronary syndromes, IVUS use was associated with higher rates of 1-year mortality [OR 2.34 (1.44–3.78), *p* < 0.001] but not the composite endpoint [OR 1.336 (0.95—1.88), *p* = 0.098].

However, in ACS there was no difference in either primary endpoint [composite endpoint: OR 0.953 (0.668—1.36), *p* = 0.792, 1-year-mortality: OR 0.748 (0.41—1.38), *p* = 0.356].

Finally, there was again no significant difference in outcome with IVUS use for patients with stented segments longer than 28 mm in either 1-year mortality [OR 1.287 (0.79—2.10), *p* = 0.314] nor MACE [OR 1.262 (0.894—1.78), *p* = 0.186].

### Propensity-matched analysis of patients receiving intravascular ultrasound and associated outcomes

Propensity matching was performed looking at all covariates that could be related to both treatment choices (IVUS or no IVUS) or treatment outcome. For completeness, we used all the covariates that were significantly different on univariate analysis.

After matching, there was excellent balance between the IVUS (treatment) and non-treatment groups. This is shown most clearly in Supplementary Figure 1, which shows the absolute mean difference between treatment groups and the Kolmogorov-Smirnov statistics.

#### Outcomes after propensity matching

**FIGURE 4:**
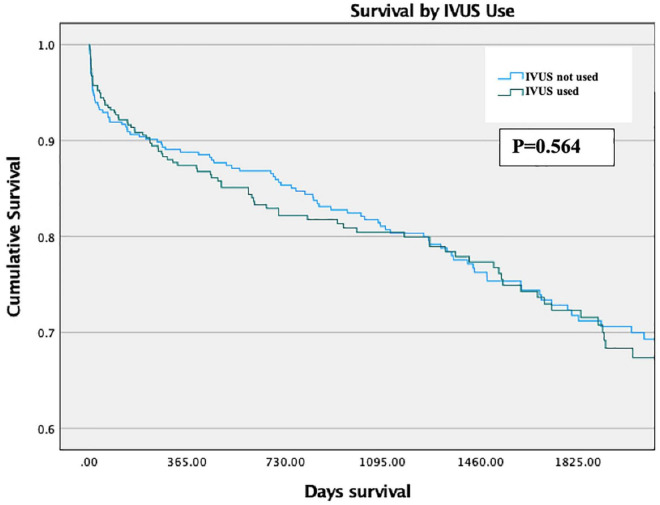
Survival by IVUS use (propensity matched groups).

There was no significant difference in Survival between propensity-matched groups via Kaplan-Meier analysis across the length of the study period (*p* = 0.564, Figure 4). This also held true for patients with stents longer than 28 mm (*p* = 0.919).

There were higher rates of MACE in IVUS arm of the propensity-matched cohort, driven primarily by increased rates of unplanned revascularisation (Table 4). This remained the case using double-robust multivariate analysis, where IVUS was associated with higher rates of MACE [OR 1.72 (1.2–2.4), *p* = 0.003].

**TABLE 4:** Outcomes for propensity-matched patients.

Outcomes in Propensity matched patients				

All patients	All patients (*n* = 794)	IVUS not used (*n* = 397)	IVUS used (*n* = 397)	*P*
Composite death/Stroke/MI/Unplanned revasc 1 year	162 (20.4)	64 (16.1)	98 (24.7)	0.003
Death 1 year	73 (9.2)	30 (7.6)	43 (10.8)	0.11
Stroke 1 year	2 (0.3)	1 (0.3)	1 (0.3)	1
MI 1 year	29 (3.7)	14 (3.5)	29 (3.7)	0.85
Unplanned revasc 1 year	71 (8.9)	23 (5.8)	48 (12.1)	0.002
Composite CV death/MI/TLR 1 year	111 (14.0)	52 (13.1)	59 (14.9)	0.474
TLR	7 (0.9)	2 (0.5)	5(1.3)	0.451
Stent thrombosis	4 (0.5)	0 (0)	4 (1)	0.124

## Discussion

Despite the growing evidence from randomized trials that IVUS improves cardiovascular outcomes across an array of patient populations, use in the UK remains low. These trials contained a significant proportion of patients with a history of previous PCI. Our study aimed to look at contemporary real-world use in the era of second generation drug-eluting stents to assess the patient, lesion and operator characteristics associated with IVUS use in addition to adjusted analyses of IVUS effect. Our analysis showed two main findings:

1. Operator characteristics are more important than patient characteristics in choosing who receives IVUS
2. Likely due to the large selection bias, there was no improvement in outcomes with IVUS use in this study

### Operator characteristics are more important than patient characteristics in choosing who receives intravascular ultrasound

Outside of a trial population, it is unsurprising that with time and resource constraints there is selective deployment of IVUS technology. Our patient population was confined to those who had never undergone PCI. There were two reasons for this. The first was that we wanted to scrutinize PCI as practiced at our institution that would not be affected by practice from other institutions. The second, and most important reason, was that we wanted to examine the effect of IVUS on the stent that was implanted with IVUS assistance, without the potential confounding of previous metalwork in the coronary tree.

The patient characteristics are significantly different between the groups. Patients who underwent IVUS-guided PCI were older, more likely to be ex-smokers and to be diagnosed with hypercholesterolemia, diabetes, hypertension and chronic kidney disease. This translated into the calcific nature of the coronary lesions—although we do not have direct markers of calcium in our data, patients who underwent IVUS were significantly more likely to need procedural devices including microcatheters, and calcium modification devices such as rotablation or shockwave lithotripsy. In multivariate analysis, the use of calcium devices was associated with an almost five-fold increase in the odds of IVUS use.

Similarly, patients requiring IVUS were less likely to be suffering an acute coronary syndrome and had fewer concomitant markers of acute cardiovascular compromise, for example having lower mean lactate levels. None of these results are surprising either at an empirical or evidence-based level. Almost all observational data shows a similar bias in operators toward this kind of patient population where stent malapposition is more likely, such as a recent study of over 100 000 patients in the United States (14). As a dedicated heart attack center in the UK, 77% of our ACS patients require primary PCI for STEMI. The observational evidence does not suggest an overwhelming benefit of IVUS use in this type of population (15–17). In fact over-expansion of a stent in a thrombotic lesion can lead to distal embolization and microvascular obstruction.

However, a more important finding was that the strongest predictor of IVUS use in our patient population was the year that the consultant left medical school. In multivariate analysis, the odds of undergoing IVUS were almost 15-fold higher if the consultant in charge graduated from medical school after 2000 compared to before 1990. As mentioned before, IVUS use is low in the UK and the USA compared to Asian countries with similar GDP per capita. There are a number of postulated reasons for this, ranging from cost and different reimbursement patterns to fears surrounding increased complication rates, through to a lack of training of interventional cardiologists. Over half of interventional trainees in the United States report limited confidence and training with IVUS (18).

The findings of our physician characteristics that were associated with IVUS use, such as generation and procedural numbers, tally with previous data on the subject that show that both a physician’s generation and patient numbers are associated with being an early adopter of technology (19). In order to maximize IVUS use in the correct patients, facilitating IVUS use for trainees and putting extra resources into training older and lower-volume operators may be a successful strategy.

The overall operator familiarity with IVUS can also affect the outcomes of patients when comparing image-guided with angiography-only PCI. Previous studies have noted the paradoxical relationship between IVUS use and patient outcomes on both an individual and population level. Operators in centers with high levels of intracoronary imaging become reliant on this technology and thus the outcomes of patients who undergo angiography-only PCI are worse because the operators are unfamiliar with the technique (20). The opposite is true in this case—our operators use IVUS in only 4.3% of cases, well below the 33% that would qualify a center as having low intracoronary imaging rates in the study above. Therefore, the operators are likely performing the angiography technique that is more comfortable and reliable, potentially leading to reduced differences between the study arms. However, it must be noted that, in clinical practice, the best operators are those who can accurately size a vessel using both angiography and intracoronary imaging modalities.

### There was no improvement in outcomes with intravascular ultrasound use in this study, probably due to the large selection bias and the high-risk population

The evidence base for IVUS use is strong and based on both observational data and numerous RCTs in the contemporary DES era. These trials have studied IVUS use in a variety of different scenarios. The first tranche examined medium-term outcome measures in patients with complex coronary disease, with the exception of the all-comers ULTIMATE trial. A meta-analysis synthesizing these trials showed that at 14 months mean follow-up, IVUS-guided PCI was associated with lower rates of cardiovascular mortality, target lesion revascularization and myocardial infarction (3).

The ULTIMATE trial (4) looked at all-comers and found significantly fewer instances of stent thrombosis and target vessel revascularization in the IVUS arm at 3 years follow-up. Further support for the benefit of IVUS at longer time points came in the shape of IVUS-XPL, which followed up patients for 5 years and demonstrated lower rates of major adverse cardiac events (5). These included death, target vessel MI and target-vessel revascularization. Finally, team members of the IVUS-XPL trial published a pooled analysis of the IVUS-XPL and ULTIMATE trials looking at patients who had ≥ 28 mm of stent inserted. There were significantly lower levels of MACE at 3 years, as reported in IVUS-XPL, although no reduction in cardiac death across 2,577 such patients and this was driven principally by TLR (6). Finally, a large-scale meta-analysis examining 31 trials, both randomized and observational trials, demonstrated that MACE was lower with IVUS use, although mortality was significantly reduced only when observational studies were included (7).

It was on this background that we began looking at patients without prior PCI. Each trial had a significant proportion of patients who had undergone prior revascularization. The lowest proportion of such patients was in IVUS-XPL, at 11%, and the greatest was almost 48% (15). The ULTIMATE trial was near the middle of this range at 18.7%. In no analysis did we find any significant improvements in patient outcomes with IVUS use, and in fact there was some suggestion of higher rates of unplanned revascularization. The same held true for patients with stents longer than 28 mm—a cut-off chosen in line with the IVUS-XPL trial and subsequent pooled analysis with ULTIMATE.

The lack of benefit shown with IVUS use likely due to the large selection bias in patient choice, as well as the high-risk nature of our population. It seems implausible that patients with IVUS-guided PCI would be more likely to require revascularization. Secondly, our mortality rate is significantly higher than other all-comers trials such as ADAPT-DES (21) which probably reflects our status as a receiver of high-risk ACS patients for the region, as shown by a cardiac arrest rate of almost 8%. Furthermore, the end-goal of IVUS use appropriate stent expansion that may require multiple procedural elements and checks, which may well be harder to accomplish in the setting of ACS. This highlights how limited this study is at assessing outcomes due to its observational design, and the fact that IVUS use is driven by all of patient, lesion and operator characteristics.

In addition, although the propensity matching was technically very good, propensity matching is only as good as the fields that are inputted. Furthermore, the propensity-matched numbers were underpowered for outcome analysis. The data inputted is largely what is found in any interventional cardiology database, but which data is accessible is not necessarily the same as which data is optimal. There is no way to propensity match a physician’s intuition for a patient, or how robust the patient looks in clinic. We can try to compensate with metrics including age, weight and important comorbidities, as we have done here, but it is never complete. This is the principal reason why RCTs exist, to exclude biases that we do not know even exist.

Therefore, the firm points that we can make in this paper are that patients who are undergoing their first PCI with a higher burden of comorbidity, in an elective setting, with lesions that are more calcified, being proceeded upon by more recently graduated cardiologists, are more likely to have IVUS used. As far as we can tell from our data, these patients are unlikely to fare better or worse compared to patients treated without IVUS when we control for these factors. We know that in other population settings, such as long stents implanted in patients with previous percutaneous revascularization, patients have better clinical outcomes with IVUS-guided angioplasty.

The strongest conclusion of this paper is that it is imperative to train more cardiologists to be comfortable with IVUS use in the UK, in order to use IVUS appropriately and in the settings that have shown to be beneficial by randomized controlled trials. The data from observational studies such as this are too confounded to suggest that there is no benefit to using IVUS in PCI-naïve patients.

## Data availability statement

The original contributions presented in this study are included in the article/Supplementary material, further inquiries can be directed to the corresponding author.

## Ethics statement

Ethical review and approval was not required for the study on human participants in accordance with the local legislation and institutional requirements. Written informed consent for participation was not required for this study in accordance with the national legislation and the institutional requirements.

## Author contributions

AT: concept, data collection, statistical analysis, manuscript preparation, and text writing. VP: concept, manuscript preparation and text writing, edits, and statistical advice. Both authors contributed to the article and approved the submitted version.

## Supplementary material

Supplementary Figure 1 Propensity matching statistics.
