# Supplementary figures and images for: Real-world intravascular ultrasound (IVUS) use in percutaneous intervention-naïve patients is determined predominantly by operator, patient, and lesion characteristics
Source: Front Cardiovasc Med. 2022 Nov 8;9:974161. doi: 10.3389/fcvm.2022.974161 (PMC9678943; doi:10.3389/fcvm.2022.974161)

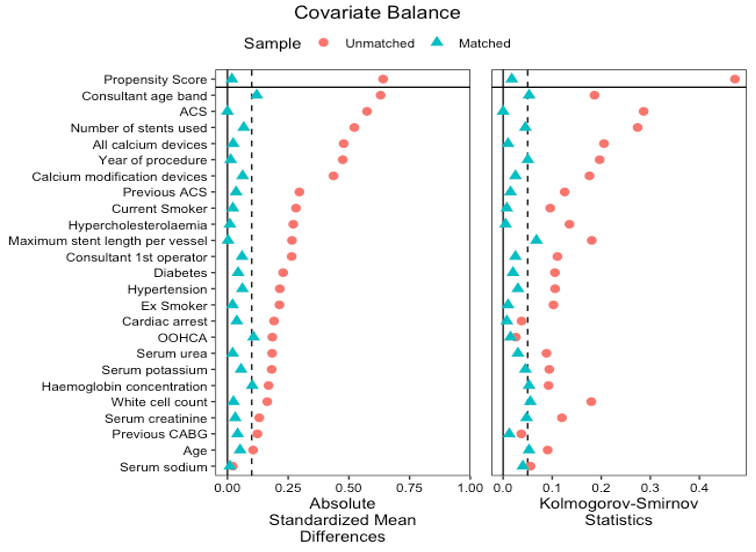

Supplement: Supplementary Figure 1 — Propensity matching statistics. [file Image_1.png]
